# How Can We Improve the Detection of Alpha1-Antitrypsin Deficiency?

**DOI:** 10.1371/journal.pone.0135316

**Published:** 2015-08-13

**Authors:** Ilaria Ferrarotti, Beata Poplawska-Wisniewska, Maria Teresa Trevisan, Janine Koepke, Marc Dresel, Rembert Koczulla, Stefania Ottaviani, Raffaele Baldo, Marina Gorrini, Giorgia Sala, Luana Cavallon, Tobias Welte, Joanna Chorostowska-Wynimko, Maurizio Luisetti, Sabina Janciauskiene

**Affiliations:** 1 Department of Molecular Medicine, Pneumology Unit, Fondazione IRCCS Policlinico San Matteo, Pavia, Italy; 2 Department of Genetics and Clinical Immunology, National Institute of Tuberculosis and Lung Diseases, Warsaw, Poland; 3 Laboratorio Analisi, Ospedale G. Fracastoro, S. Bonifacio, ULSS20, Verona, Italy; 4 Division of Pulmonary Diseases, Department of Internal Medicine, German Center for Lung Research (DZL), Philipps-Universität Marburg, 35037, Marburg, Germany; 5 Department of Respiratory Medicine, Hannover Medical School, Biomedical Research in End stage and Obstructive Lung Disease Hannover (BREATH), German Center for Lung Research (DZL), 30625, Hannover, Germany; RWTH Aachen, GERMANY

## Abstract

The Z deficiency in α1-antitrypsin (A1ATD) is an under-recognized condition. Alpha1-antitrypsin (A1AT) is the main protein in the α1-globulin fraction of serum protein electrophoresis (SPE); however, evaluation of the α1-globulin protein fraction has received very little attention. Serum Z-type A1AT manifests in polymeric forms, but their interference with quantitative immunoassays has not been reported. Here, 214 894 samples were evaluated by SPE at the G. Fracastoro Hospital of Verona, Italy. Patients with an A1AT level ≤ 0.92 g/L were recalled to complete A1ATD diagnosis. In parallel, to qualitatively and quantitatively characterize A1AT, sera samples from 10 PiZZ and 10 PiMM subjects obtained at the National Institute of Tuberculosis and Lung Diseases in Warsaw, Poland, were subjected to non-denaturing 7.5% PAGE and 7.5% SDS-PAGE followed by Western blot. Moreover, purified A1AT was heated at 60°C and analyzed by a non-denaturing PAGE and 4–15% gradient SDS-PAGE followed by Western blot as well as by isolelectrofocusing and nephelometry. A total of 966 samples manifested percentages ≤ 2.8 or a double band in the alpha1-zone. According to the nephelometry data, 23 samples were classified as severe (A1AT ≤ 0.49 g/L) and 462 as intermediate (A1AT >0.49≤ 1.0 g/L) A1ATD. Twenty subjects agreed to complete the diagnosis and an additional 21 subjects agreed to family screening. We detected 9 cases with severe and 26 with intermediate A1ATD. Parallel experiments revealed that polymerization of M-type A1AT, when measured by nephelometry or isolelectrofocusing, yields inaccurate results, leading to the erroneous impression that it was Z type and not M-type A1AT. We illustrate the need for confirmation of Z A1AT values by “state of the art” method. Clinicians should consider a more in-depth investigation of A1ATD in patients when they exhibit serum polymers and low α1-globulin protein levels by SPE.

## Introduction

α1-Antitrypsin (A1AT) is encoded by the protease inhibitor (Pi) locus on chromosome 14q32.1, as a part of a gene cluster called the SERPIN supergene [1]. The nomenclature used to identify A1AT variants of the allelic system called “Pi-system” was developed during the 70s, based on the migration velocity of A1AT variants in an electric field. The position of the migrating proteins is identified by a letter, where PiM indicates medium (normal), PiF fast, PiS slow and PiZ very slow [2]. The enormous number of A1AT variants that have been identified up to now are classified into four major categories for clinical purposes: normal, deficiency, null and dysfunctional [3,4].

The Z variant of A1AT, which differs from the normal M variant in the substitution of Glu342 with Lys [5], is the most prevalent A1AT deficiency (A1ATD) variant and is related to the significant risk for developing early onset chronic obstructive lung disease (COPD) and liver disease at any age. The prevalence of PiZZ A1ATD is 1 in 1,500–5,000 individuals, which suggests that approximately 100,000 subjects in western countries are affected by this mutation [6,7]. In spite of the efforts made over the last two decades to improve detection of A1ATD individuals, data from the two largest registries- the Alpha One International Registry and the Alpha-1 Foundation Research Network Registry-indicate that less than 5% of estimated A1ATD subjects have been identified [7–9].

Several strategies are being employed to improve A1ATD detection rates [10]. Mass screenings have been performed in cohorts from the general population, students, newborns or blood donors. This approach is obviously limited by the high costs that normally hamper large scale programs. According to ATS/ERS recommendations [11], all patients with COPD and asthma (not fully reversible after bronchodilator therapy) should be tested for A1ATD. This latter strategy, also known as the case-finding strategy, results in a much higher A1ATD detection rate than mass screening programs [10]. However, ATS/ERS recommendations have been largely disregarded. Only 18–25% of physicians in Germany and Italy, who took part in the survey, tested all COPD patients for A1ATD [12, 13]. An alternative strategy suggested by the ATS/ERS guidelines [11] is the so-called targeted detection, which tests specific categories of subjects: those with early onset emphysema, emphysema prevalent in the lower lobes or familial clustering of COPD, and first degree relatives of subjects diagnosed with severe or intermediate A1ATD. Testing for subjects with an absent or reduced α1-globulin band on routine serum protein electrophoresis (SPE) is suggested as well.

Severe PiZZ A1ATD is associated with about 90% lower levels of plasma A1AT (normal levels are 1–2 g/L) that arise not from the lack of protein synthesis, but rather from its intracellular polymerization [7]. Novel studies provide evidence that extra-hepatic polymerization of Z A1AT occurs [14]. A mouse monoclonal antibody, ATZ11, recognizing Z-type polymers of A1AT, has been widely used for the recognition of A1AT deficiency in ELISA procedures [15, 16]. Non-Z carriers were found to have very low levels or a total lack of plasma A1AT polymers, which explains the high sensitivity and specificity of the ATZ11-based ELISA system used for the detection of Z carriers in earlier studies [16]. Recently, researchers have developed a new conformation-specific monoclonal antibody (2C1) that specifically recognizes polymers formed by A1AT [17]. This antibody was used to quantify plasma polymers and confirmed that circulating polymers of A1AT are present in all Z A1ATD individuals and that they originate from the liver.

The information described above, encouraged us to conduct a pilot study addressing the potential of routine SPE, as a tool to improve A1ATD screening and detection. Because polymeric forms of Z A1AT are found in serum, the next important question was whether these forms interfere with A1AT quantification when performing routine immunoassays.

## Materials and Methods

### Routine serum protein electrophoresis (SPE)

This study was performed on samples previously collected from January 2001 to December 2013. This institutional database includes patients who gave written informed consent for use of their medical data and biological samples in future research. The records are accessed in a fully anonymized and de-identified manner. Routine SPE (CAPILLARYS 2, capillary electrophoresis system, Sebia, Inc. and horizontal agarose gel electrophoresis system, Bio-Rad laboratories) was performed at the Clinical Chemistry facility of the G. Fracastoro Hospital Health District 20 of Verona, Italy. Whereas overall the high-resolution available from capillary electrophoresis system is an advantage against agarose gel electrophoresis, it also has some disadvantages. In the capillary electrophoresis the lower and upper ranges for α1-globulin are twice as high as the ranges for the agarose gel-based systems. The increase in α1-globulin likely reflects the increased ability of capillary electrophoresis to detect both α1-lipoprotein and α1-acid glycoprotein (orosomucoid) compared agarose gels. Moreover, unlike capillary electrophoresis system, agarose gels can separate proteins larger than 600,000 Da. When SPE revealed a α1-globulin band percentage < 2.8% of the total protein content or when the double band, that usually accounts for heterozygous of alpha1-antitrypsin, was detected in the α1-zone, the sample was submitted to quantitative A1AT determination. The concentration of total protein was determined with the biuret method and the Roche/Hitachi Cobas c systems. The total protein concentration was multiplied by the relative SPE α1-globulin total density percentage to yield the final α1-globulin peak concentration. Based on the results, a sample was submitted to complete A1ATD testing, including isoelectric focusing, genotyping and sequencing (if necessary) [18]. Subjects were selected on the basis of a “bottom-to-top” ranking (individuals with the lowest A1AT serum level were recalled first). The Institutional Review Board (IRB) of the Fondazione Policlinico IRCCSS an Matteo Hospital approved the complete A1AT testing in 2005 prior to analysis. Patient identities were removed and patient records were used anonymous.

### Quantitative determination of A1AT by Nephelometry

The A1AT quantitative analysis was performed using an immune nephelometric method (Immage 800 Immunochemistry System, Beckman-Coulter, USA) with commercially available reagents containing goat anti-human A1AT antibody (Beckamn-Coulter, USA). The nephelometer automatically dilutes analysed samples 1:216 to achieve optimal antigen-antibody equilibrium in the assay. The normal range of the instrument for A1AT in serum samples is 88–174 mg/dL with a cutoff value of 120 mg/dL [19].

### Phenotyping of A1AT by Isoelectrofocusing (IEF)

A1AT phenotype analysis was performed by isoelectrofocusing ready-to-use agarose gels with specific immunological detection using the Hydragel 18 A1AT Isofocusing kit with the semi-automatic Hydrasys System (Sebia) as described previously [20]. Phenotypes of serum A1AT samples were determined by comparing their migration patterns with control samples.

### Determination of A1AT molecular profiles in serum of PiMM and PiZZ subjects

Serum samples from COPD individuals classified as PiMM (n = 10) or PiZZ (n = 10) were obtained during routine clinical laboratory analyses at the National Institute of Tuberculosis and Lung Diseases in Warsaw, Poland. The study was approved by the IRB of the National Institute of Tuberculosis and Lung Diseases in Warsaw (01–138). Every patient gave written informed consent for collection and use of serum samples for this study. Samples were analyzed by7.5% native polyacrylamide gel electrophoresis (PAGE) and by 7.5% sodium dodecyl sulfate (SDS)-PAGE electrophoresis under non-reducing conditions, which leaves disulphide bonds intact. Electrophoretically separated proteins were transferred to a polyvinylidene fluoride membrane (Millipore; Millipore Corporation; Bedford, MA) using the semidry blot electrophoretic transfer system. Western blots were accomplished using mouse monoclonal anti-A1AT (1:500; B9, sc-59438, SantaCruz, USA), mouse monoclonal antibody, ATZ11, which recognizes both polymerized and elastase-complexed forms of A1AT (1:100, our own) and mouse monoclonal 2C1 that specifically recognizes A1AT polymeric forms (1:100, Catalog #: HM2289, Hycult Biotech, The Netherlands). The immune-complexes were visualized with secondary horseradish peroxidase-conjugated swine anti-rabbit or rabbit anti-mouse (1:10 000, Dako, Denmark) antibodies and the ECL Plus Western blot detection kit (Amersham Biosciences; Buckinghamshire, UK).

### Preparation and analysis of polymerized M A1AT *in vitro*

A vial of lyophilized A1AT (Prolastin) was re-suspended as recommended in the manufacturer’s leaflet. Afterwards, the product was diluted with phosphate-buffered saline solution (PBS), pH 7.4, to generate a 2 mg/ml A1AT stock solution and heated at a fixed temperature of 60°C with vibration (frequency of 300 min-1, HLC Heating ThermoMixer, USA) for different time points (from 5 to 240 min). At each time point, aliquots of A1AT were immediately analyzed on 4–15% linear gradient SDS-PAGE without sample heating and on non-denaturing 7.5% PAGE, followed by a Western blot using rabbit polyclonal antibody anti-A1AT (1:800; Dako; Glostrup, Denmark). In parallel, A1AT samples were assayed by nephelometry and IEF. The IEF analysis of heat-modified A1AT was performed with Hydrasys 2 (Sebia) with the respective Hydragel 18 A1AT isofocusing kit and reagents (Sebia) in the clinical laboratory of the Marburg University Hospital, Germany.

## Results

### Data from routine SPE analysis

The SPE analysis was performed on 214,894 samples, among which 85,200 were performed by high-resolution agarose gel electrophoeresis (AGE) and 129,694 by capillary zone electrophoresis (CZE). In total, 966 samples were subjected to further analysis as they resulted in ≤ 2.8 percentage of α1-globulin band or a double band was detected in the α1-zone. According to the nephelometry data, 348 out of 966 subjects (79 with α1-globulin percentage ≤ 2.8, and 269 with α1-globulin percentage >2.8) had a serum A1AT level above 1.13 g/L; for those the A1ATD diagnostic flow chart was discontinued. On the other hand, 23 samples were in the severe (A1AT ≤ 0.49 g/L) and 369 in the intermediate (A1AT >0.49≤ 0.92 g/L) A1ATD zone. Finally, 226 subjects had a serum level of A1AT above the intermediate A1ATD threshold (A1AT >0.92 g/L [21]), but below the decisional cut-off (Table 1).

**Table 1:** Summary of the nephelometric measurments of A1AT concentration in 966 samples analysed using routine serum protein electrophoresis (SPE) methods.

α1-globulin fraction	SPE method	A1AT concentration determined by nephelometry (g/L)			
		*≤ 0*.*49*	*> 0*.*49 ≤ 0*.*92*	*> 0*.*92 ≤ 1*.*13*	*> 1*.*13*
**% ≤ 2.8**	CZE	12 (4.56)	157 (59.70)*	69 (26.24)	25 (9.50)
	AGE	9 (4.84)	99 (53.23)	24 (12.9)	54 (29.03)
**% > 2.8**	CZE	2 (0.41)	111 (22.75)	132 (27.05)	243 (49.79)
	AGE	0	2 (7.00)	1 (3.50)	26 (89.65)

CZE-capillary zone electrophoresis; AGE-agarose gel electrophoresis;

* mean (SD)

### Assessment of SPE accuracy

We assessed the SPE (CZE and AGS methods) accuracy in predicting plasma A1AT concentration using ROC statistics (Table 2). The densitometric analysis was performed based on the total serum protein concentration with a ROC analysis. The comparison between ROC curves evidenced that the threshold calculation of the α1-globulin percentage/total protein is preferable. The optimal threshold for suspecting A1ATD according to the Youden index provided a cut-off of % α1-globulin/ total protein at 2.36 or 1.61g/L, which has a sensitivity of 85.3% or 92.6 and a specificity of 76.0% or 92.5, for CZE and AGE, respectively. The optimal threshold for suspecting severe A1ATD according to the Youden index provided a cut-off of % α1 globulin/ total protein at 2.07 or 1.12g/L, which has a sensitivity of 92.9% or 88.9 and a specificity of 65.6% or 87.9, for CZE and AGE, respectively.

**Table 2:** Thresholds for suspicion of severe or intermediate A1ATD according to SPE.

A1AT (g/L)	SPE	% α1-globulin				% α1-globulin/total protein (g/L)				P
cut-off	method	best value	sensibility	specificity	AUC	best value	sensibility	specificity	AUC	
**1.13**	**CZE**	≤3.40	92.5	63.8	0.854	≤2.36	85.3	76.0	0.876	p = 0.02
	**AGE**	≤2.00	89.6	93.7	0.952	≤1.61	92.6	92.5	0.962	
**0.49**	**CZE**	≤2.70	78.6	77.7	0.836	≤2.07	92.9	65.6	0.855	p = 0.03
	**AGE**	≤1.40	77.8	90.8	0.895	≤1.12	88.9	87.9	0.923	

AGE-agarose gel electrophoresis; CZE-capillary zone electrophoresis.

Patients with a serum A1AT level ≤ 0.49 g/L were recalled, when possible, to complete A1ATD diagnosis. Seven out of 14 (50%) recalled patients, returned for further testing. We enlarged the recall to include subjects with a serum A1AT level ≤ 0.92 g/L, which is the threshold value for intermediate A1ATD [21]. Subjects were selected on the basis of a “bottom-to-top” ranking (individuals with the lowest A1AT serum level were recalled first). Among the subjects that were recalled to complete diagnosis, 20 out of 307 (9.7%) agreed and completed the A1ATD diagnosis. In addition, 21 subjects spontaneously agreed to perform the family screening (Table 3). When also considering subjects submitted to the family screening, we detected 9 new subjects with severe and 26 with intermediate A1ATD.

**Table 3:** Summary of A1ATD detection using the routine SPE strategy.

Genotype	Data from routine SPEP		
	AAT ≤0.49 g/L	AAT 0.49–0.92 g/L	Family Screening
PI*MM			**6**
PI*MS		**1**	
PI*MZ		**5**	**11**
PI*MM_**procida**_		**3**	**1**
PI*MM_**malton**_		**1**	**1**
PI*MP_**lowell**_			**2**
PI*SZ	**2**	**1**	
PI*SP_**Lowell**_		**1**	
PI*SM_**malton**_	**1**		
PI*ZZ	**2**		
PI*ZN_**vestenanova**_ ^1^		**1**	
PI*ZM_**procida**_	**1**		
PI*ZP_**Lowell**_	**1**		

^1^N_vestenanova_ is a novel variant (Ala325GCA/ProCCA), not reported previously.

### Electrophoretic characterization of serum A1AT in PiMM and PiZZ subjects

**Fig 1:**
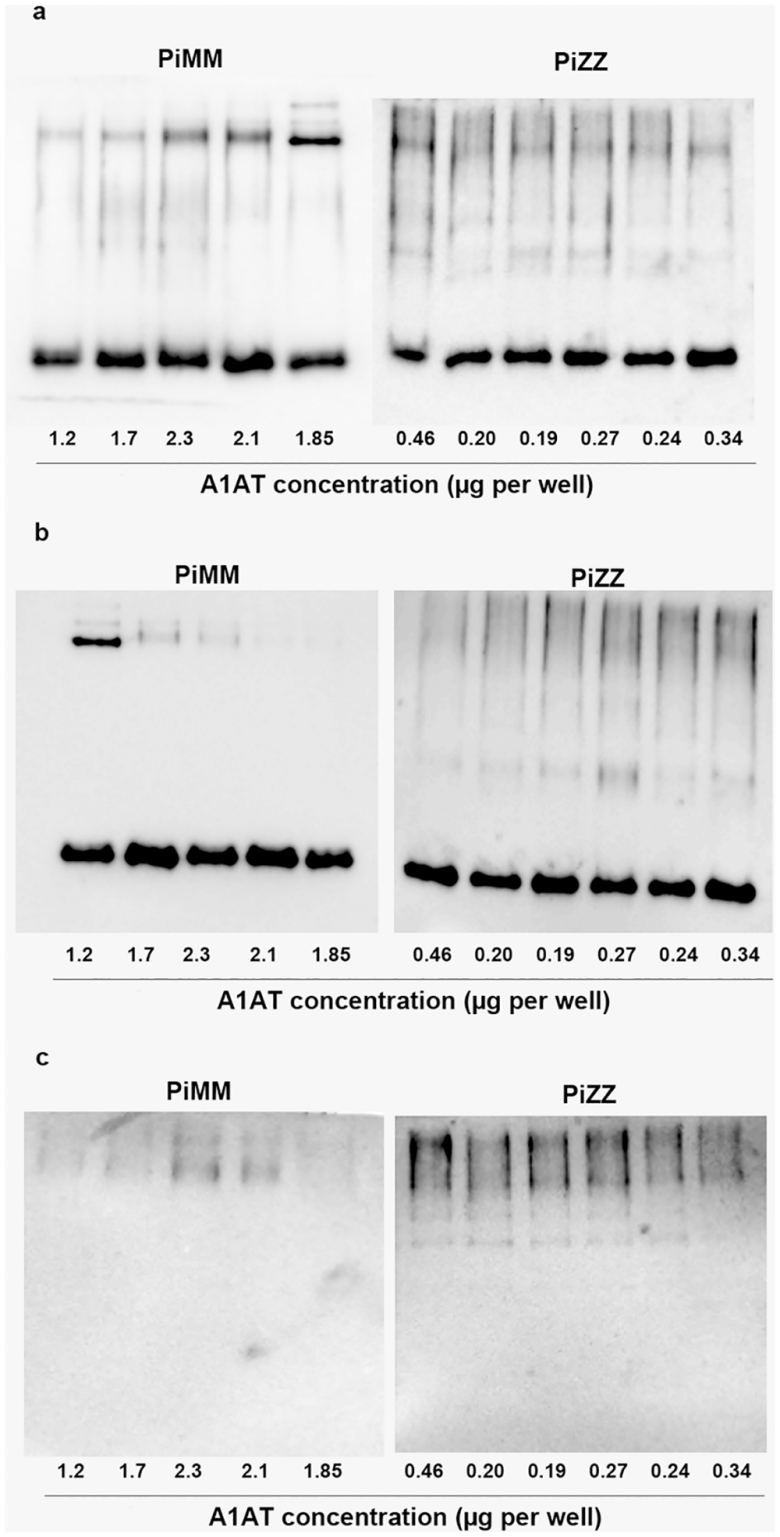
Representative immune blot analysis of A1AT in MM and ZZ serum. The serum concentration of A1AT was determined using routine nephelometric analysis. Equal volumes of diluted serum samples (1:100 in sterile 0.15M NaCl) were loaded and separated on the 7.5% PAGE, and Western blots were performed using specific anti-A1AT antibodies: a) mouse monoclonal against human A1AT; b) mouse monoclonal antibody, ATZ11, recognizing Z-type polymers of A1AT and c) mouse monoclonal 2C1 specific against A1AT polymers. Relative A1AI concentration in the loaded sample per well is indicated at the bottom of the gels.

To qualitatively characterize A1AT in serum of PiZZ and PiMM subjects, equal amounts of diluted samples were subjected to non-denaturing 7.5% PAGE followed by Western blot. The immunoblot patterns using different anti-A1AT antibodies indicated that PiMM and, in particular, PiZZ serum comprises a mixture of differently charged molecular forms of A1AT (Fig 1a–1c). Either because of reduced immunoreactivity or a high degree of polymerization, the immunoprecipitates of A1AT from PiZZ serum continued to stain and thus did not allow a precise characterization of the molecular profile. To confirm that PiZZ serum contains significant amounts of polymers, we used specific ATZ11 and 2C1 monoclonal antibodies, which react with A1AT polymers. As illustrated in Fig 1b and 1c, each PiZZ serum sample contains polymeric forms of A1AT, whereas few PiMM samples also showed any degree of A1AT polymerization.

**Fig 2:**
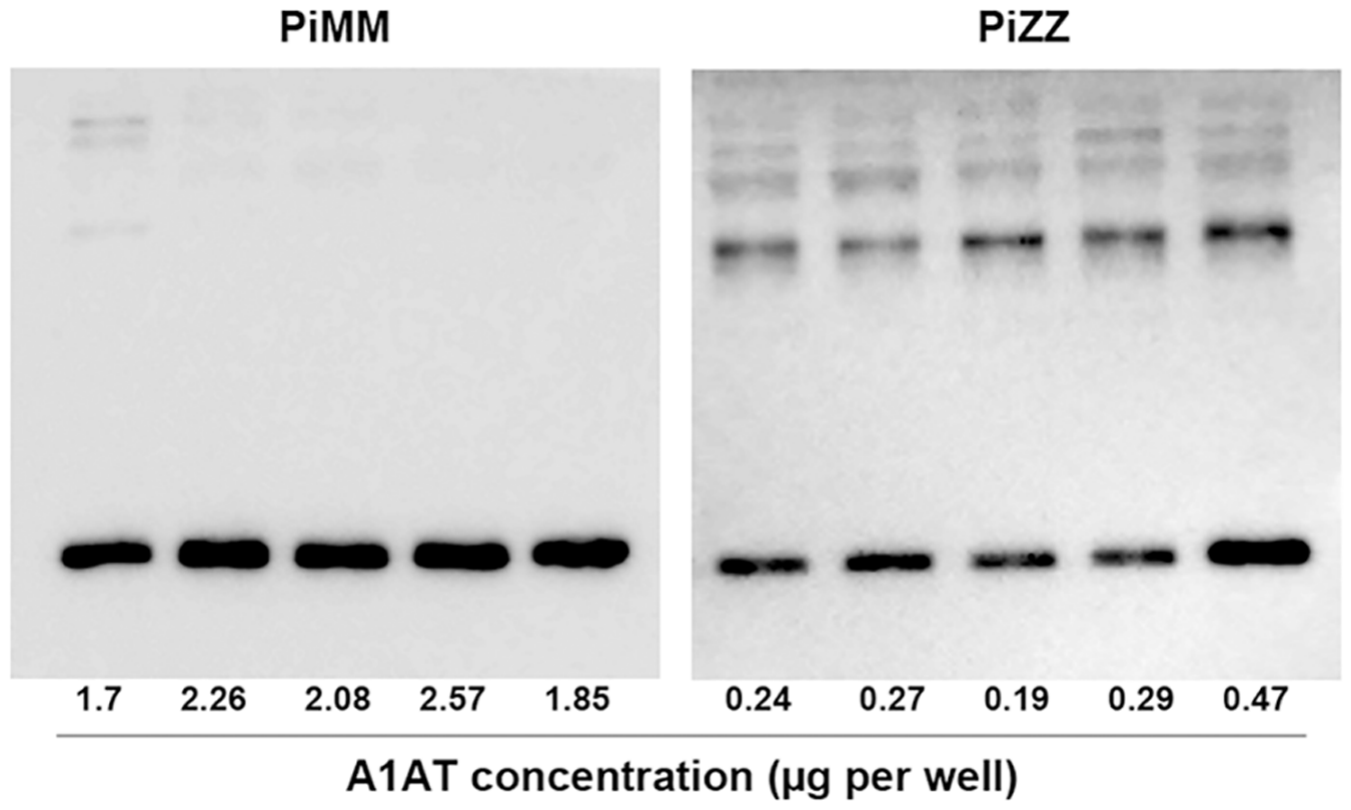
Molecular analysis of serum A1AT form in patients with the MM or ZZ genotype. Serum concentrations of A1AT were determined using routine nephelometric analysis. Equal volumes of diluted serum samples (1:100 in sterile 0.15M NaCl) were loaded and separated on the 7.5% SDS-PAGE (without denaturing agents), and Western blots were performed using specific anti-A1AT antibodies: a) mouse monoclonal against human A1AT; b) mouse monoclonal antibody, ATZ11, recognizing Z-type polymers of A1AT and c) mouse monoclonal 2C1 specific against A1AT polymers. Relative A1AI concentration per well in the loaded serum sample is indicated at the bottom of the gels.

The same serum samples were subjected to 7.5% SDS-PAGE (without denaturing agents) followed by Western blotting using the same monoclonal anti-A1AT antibody as in Fig 1. Comparison of the immunoblots of PiMM with PiZZ serum revealed that PiMM samples typically have one distinct band of the A1AT monomer (molecular size about 54 kDa) whereas PiZZ serum samples contain a band similar to the A1AT monomer, but also various sized polymers of A1AT (Fig 2a–2c). Based on nephelometry data, PiMM and PiZZ samples contain significantly different concentrations of A1AT protein. Hence, gel loading with the equal volumes of precisely diluted PiMM and PiZZ samples will result in very different concentrations of A1AT. Interestingly, despite significant differences in PiMM and PiZZ A1AT concentrations, after taking into account A1AT polymers, the immune-precipitates of A1AT in all samples looked relatively similar (Figs 1 and 2).

### Molecular profile characterization of heat-treated purified A1AT

**Fig 3:**
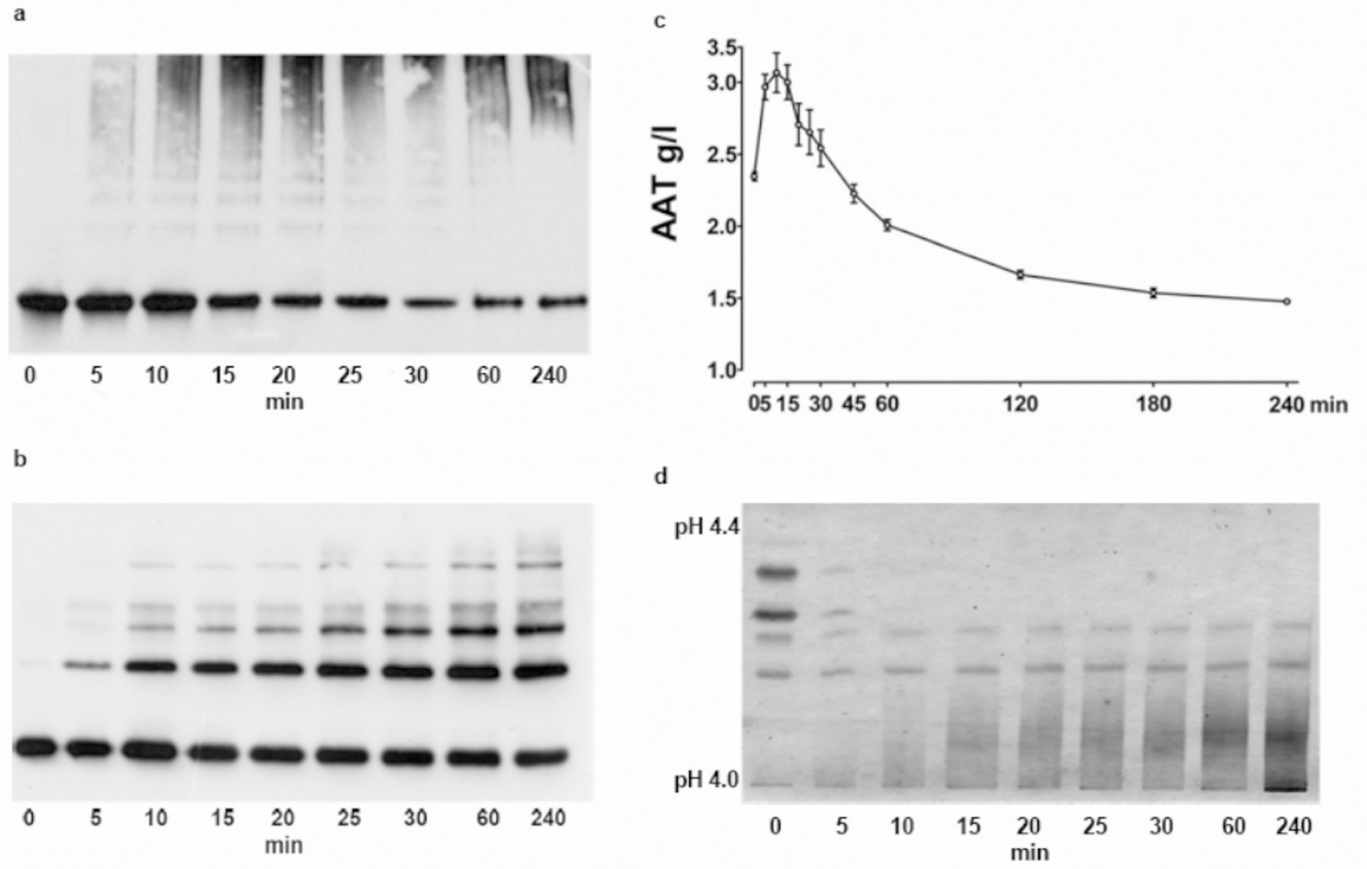
a-d. Molecular profile of heat-treated purified A1AT. **a**. Purified plasma A1AT (2.5 mg/ml) was heated at 60°C and aliquots were removed at indicated time points for analysis by a non-denaturing PAGE followed by Western blotting using rabbit polyclonal anti-A1AT antibody. **b**. For a more detailed analysis of the heat-treated A1AT a 4–15% gradient SDS-PAGE was used. This method is designed for separation of high molecular weight polymers. Electrophoretic separation of A1AT was followed by Western blot analysis with an anti-human A1AT antibody as above. The immunoblot patterns indicate that heat-treated A1AT typically comprises a mixture of a monomer and various sized polymers. The figure shows representative blots from 5 experiments. **c**. M A1AT heat-treated for 5 min exhibited a typical five band pattern in the IEF gel (lane 1). When M A1AT was heated for 15 min or longer t, the three prominent bands towards the anode were no longer detectable (lanes 2–9). Instead, a continuous smeared band occurred at the cathode (lane 3), these results paralleled those with heat-induced A1AT polymerization over time (Fig 3b). **d**. Initial concentrations of purified A1AT were 2.35 ± 0.042 mg/ml; n = 3. The same A1AT protein heated for a short period yielded a higher nephelometric signal than native protein. However, heating of A1AT for 45 min or longer, resulted in a remarkable decrease in the nephelometric signal relative to the native protein.

The results above implied that polymers of A1AT may interfere with A1AT quantitative characterization using immunological methods and encouraged us to perform an *in vitro* test. Purified plasma A1AT (2.5 mg/mL) was heated at 60°C and aliquots were removed at indicated time points for analysis by a non-denaturing PAGE followed by a western blot using rabbit polyclonal anti-A1AT antibody. As shown in Fig 3a, when A1AT was heated for 5 min it appeared as a single sharp band. After longer heating of the A1AT protein, the single band appeared lower in protein concentration than the corresponding new blurred bands in the upper part of the gel. For a more detailed analysis of the heat-treated A1AT we used 4–15% gradient SDS-PAGE designed for separation of high molecular weight polymers. Again, electrophoretic separation of A1AT was followed by Western blot analysis with an antibody against human A1AT as above. The results revealed that heat-treatment of A1AT samples up to 120 min leads to the formation of increasing amounts of polymeric forms of A1AT. Prolonged heating (up to 240 min) had no additive effect on A1AT polymerization (Fig 3b). The patterns of immunoblots indicated that heat-treated A1AT typically comprises a mixture of a monomer and four various sized polymers.

### Heat-modified M A1AT is not detectable by isoelectric focusing (IEF)

M A1AT purified and heated for 5 min exhibited a typical five band pattern in the middle of the IEF gel (Fig 3c), lane 1). However, when M A1AT was heated for 15 min or longer, the three prominent bands close to the anode were no longer detectable (Fig 3c), lane 2–9). Instead, a continuous smeared band occurred at the cathode (Fig 3c, lane 3), which paralleled with heat-induced A1AT polymerization over time (Fig 3b).

### Polymerization of purified M A1AT affects protein quantification by nephelometry

As illustrated in Fig 3d), nephelometric analysis revealed a purified A1AT concentration of 2.35 ± 0.042 mg/ml; n = 3. Initially, the same heat-treated A1AT (60°C for 30 min) yielded a higher nephelometric signal than native protein. However, heating of A1AT for 45 min or longer, resulted in a remarkable decrease in nephelometric signal relative to native protein. The reduction in the concentration of A1AT paralleled time-dependent heat-induced A1AT polymerization (Fig 3b).

## Discussion

A1ATD still remains an under-recognized and undertreated condition, and therefore continues to pose a significant health threat [22]. Strategies to identify affected individuals include both population-based screening and targeted detection. Mass strategies have the advantage of unbiased, prejudice-free detection, thus they potentially include also healthy or asymptomatic individuals carrying A1ATD genotypes [10]. To date, the largest population-based screening studies have been conducted by O'Brien et al. in Oregon [23] and Sveger in Sweden [24], as neonatal screenings. These strategies incur extremely high costs and necessitate complicated organizational efforts. Moreover, strategies including screening the general population or large unselected cohorts yield lower A1ATD detection rates than targeted detection or case-finding strategies. Hence, most studies are undertaking targeted detection strategies and employ a variety of testing methods.

Strategies to enhance A1ATD detection in targeted studies include awareness campaigns and simple testing techniques, such as evaluation of dried blood spots. The diagnosis of Z-type A1ATD relies on the demonstration of a low serum concentration of A1AT, the detection of A1AT protein variants by protease inhibitor (PI) typing and the detection of mutations in both copies of SERPINA1, the gene encoding A1AT. Electrophoresis can also be used to identify A1ATD based on the absence of a normal α1-globulin band. An abnormal A1AT electrophorectic pattern is seldom diagnostic by itself but, rather raises a flag that for the testing is required. Encouraging diagnostic results were observed with a routine SPE technique utilized by Slev and colleagues [25]. From estimates of PiMZ and PiZZ phenotype prevalence, the authors calculated that one PiZZ A1ATD case can be identified in approximately every 31 sample with α1-globulin concentrations of less than 0.21 g/dL.

In clinical laboratories, SPE is mainly performed by AGE and automated CZE systems. The CZE system is often suggested as an alternative for conventional AGE, because it allows fast protein separation with good resolution and high sensitivity. However, regarding the detection of A1ATD, it seems that CZE has a lower specificity than AGE [26,27]. SPE uses an electrical field to separate proteins into groups based on their relative electrophoretic mobility, which depends on protein charge, size and shape. Although in routine SPE the major component of the α1-globulin protein fraction is A1AT, other proteins, including α1-acid glycoprotein and apolipoprotein A-1, also contribute to the measured α1-globulin fraction [28] and can mask A1ATD. It is also important to note that protein electrophoregrams allow only semi quantitative analyses of proteins and that a α1-globulin cut-off has not been established for the identification of patients with A1ATD. Therefore, we wanted to investigate whether routine SPE samples with a reduced α1-globulin band would be useful in detecting A1ATD.

In agreement with previous studies [26,27], we found CZE to be less effective than the AGE system in identifying the relationship between a reduced α1-globulin band and lower plasma concentration of A1AT (measured by nephelometry) (Table 1). Furthermore, we found that our cut-off of % α1 globulin/ total protein at 2.07 g/L for detecting severe A1ATD with CZE is superimposable to the value of 0.21g/dL proposed by Slev and colleagues [25]. Overall, we concluded that the detection rate of A1ATD using routine SPE techniques seems to be in concordance with the case finding programs, i.e. resulting in the identification of 2.3% cases. We also revealed several subjects with serum levels of A1AT ≤ 0.92g/L, which were affected either by severe or intermediate A1ATD. Despite the medium-low interest of participants to enroll in this project, we detected 9 new subjects with severe A1ATD. Thus, routine SPE seems to be a suitable method for quantification of the α1-globulin band and detection of A1ATD.

Secondly we wanted to evaluate putative explanations for why detection of A1ATD remains difficult with the available immunological technologies. A1AT is an acute phase protein; therefore, measuring A1AT concentration alone can be misleading. The increase in A1AT concentration due to inflammation or various types of infection can mask the presence of A1ATD [29]. On the other hand, Z-type A1AT forms polymers [15], and we think that polymers might also interfere with routine immunochemical methods. Nephelometric assays used for the quantitation of serum A1AT utilize a high-intensity light source that passes through a reaction vessel containing the antigen-antibody complexes, and a photo detector collects the light scattering signal. The reaction requires at least a bivalent immunoglobulin and an antigen with at least two epitopes. As a rule, all nephelometric assays have to operate in the presence of antibody excess. As more antigens are added, the reaction moves towards antigen excess, all free antibodies are consumed, and no precipitation occurs due to the lack of antibody. Nephelometry assays used in the clinical laboratories are adopted for the sera containing the monomeric form of A1AT protein. Therefore, the multiple polymeric forms of Z A1AT may be missed resulting in an underestimation of A1AT protein concentration. For example, as PiMM serum contains about 90% more A1AT than PiZZ serum, its immune reactivity is expected to be proportionally higher when examined by native PAGE or non-reducing SDS-PAGE electrophoresis followed by Western blotting. Comparison of the immunoreactive properties of PiMM and PiZZ samples verified that Z-type A1AT occurs in monomeric and different polymerized forms. However, despite the fact that the immunoelectrophoretic pattern of Z A1AT differs from M-type A1AT, immunoreactivity of PiMM and PiZZ serum has been found to be relatively similar. These data constitute evidence that in all likelihood these discrepancies are related to a polymerized state of Z-type A1AT.

To test this prediction we used different immunochemical methods for qualitative and quantitative characterization of purified plasma M A1AT, before and after heat-induced polymerization. As expected, purified A1AT manifested a single immunoprecipitation band with immunoblotting, whereas heat-treated A1AT changed immunoprecipitation patterns in direct relation to the heating time: a gradually weakening anodal band and occurring more pronounced bands with retarded mobility towards the cathode were observed. However, most strikingly, heat-treated M A1AT exhibited a Z A1AT pattern in IEF assays and resulted in about 50% lower concentrations when quantified by nephelometry (Fig 3d). These data constitute the first direct evidence that polymeric A1AT exhibits changes in mobility and in immunoreactivity. It is probable that polymeric A1AT exhibits more pronounced changes in electrophoretic mobility with AGE than with CZE, and a α1-globulin band does not occur. These physicochemical and immunogenic changes in polymeric A1AT might in part explain why for Z-A1ATD detection that the AGE method might be more specific than CZE.

Our data illustrate how the true concentration of A1AT might be underestimated due to polymer formation. Small changes in the antigen structure (such as a single amino acid in Z-A1AT) can affect the affinity of the antibody-antigen interaction in immunological methods, whereas retarded polymer migration may show reduced protein levels in the α1-globulin band. Further evaluation of electrophoretic and immunological methods routinely used for A1ATD is warranted. Novel studies are also needed to determine appropriate quantitative α1-globulin fraction cutoffs for electrophoretic methods.

Taken together, we believe that this pilot study, even with its experimental limitations, should alert clinicians and researchers to pay attention to the influence of Z A1AT polymers on the accuracy of quantitative and qualitative analysis of A1AT. Our study may also encourage the development of more structured routine SPE protocols in A1ATD detection programs and, thereby, provide earlier detection of A1ATD.

### Take home message

The Z deficiency of α1-antitrypsin (A1AT) is an under-recognized condition related to the significant risk of developing COPD and emphysema. A1AT is the main protein in the α1-globulin fraction of serum protein electrophoresis (SPE); however, evaluation of the α1-globulin protein fraction has received very little attention. Moreover, serum Z-type A1AT manifests in polymeric forms, but their interference with quantitative immunoassays has not been reported. We illustrate that routine SPE is a suitable method for quantification of the α1-globulin band and the detection of A1AT deficiency. We also show that polymerization of M A1AT, when measured by nephelometry or isolelectrofocusing, yields inaccurate results, leading to the erroneous impression that it was Z-type and not M-type A1AT. Clinicians should consider a more in-depth investigation of A1AT deficiency in patients when they exhibit serum polymers and low α1-globulin protein levels by SPE. The early detection of A1AT deficiency could prevent disease development.
